# Medicine, Pathology and Therapeutics

**Published:** 1896-04

**Authors:** 


### MEDICINE, PATHOLOGY AND THERAPEUTICS.

Conducted by a member of the editorial staff.

### THERAPEUTIC VALUE OF BENZOSOL.

WHITE, (*Richmond Journal of Practice*, January, 1896,) in discussing this subject, says that benzosol is a combination of the ethreal salt of guaiacol and benzoic acid ; that when administered by the mouth it is not dissolved by the juices of the stomach, but the guaiacol is liberated in the intestinal canal and is subsequently excreted by the kidneys with the benzoic acid.

White affirms that the great advantages of benzosol are its comparative freedom from odor or taste and its readiness of assimilation; that digestive disturbances very rarely occur during its administration; that no ill-effects have been observed, but that the limit of dose mentioned (a dram a day) has not been exceeded.

White then details its use in phthisis and other tubercular diseases, giving the studies of Walzer, affirming, however, that his own cases most benefited were those which manifested a marked catarrhal condition of the lungs in the early stage, and even such as were well advanced, with formation of cavities and excessive expectoration. He further testifies to the value of the drug in gastric and intestinal dyspepsia and in diabetes, relating his own experiences in considerable detail. In speaking of its effects on the latter disease, he says:

> Not only are evidences of good to be noted in the change of the condition of the urine, but the general health also responds under its favorable influence, as shown by increased appetite, facilitation of the processes of digestion and assimilation with the resulting improvement in nutrition.

---

### TREATMENT OF DIPHTHERIA AT THE HARPER HOSPITAL, DETROIT, MICH.

DR. B. S. SHURLY, (*Therapeutic Gazette*, February 15, 1896,) house physician of the Harper Hospital, describes very carefully twenty-six cases of diphtheria treated with anti-diphtheritic serum, and draws the following conclusions:

1. The cases were under constant observation from the time of admission to discharge; this being in contradistinction to cases observed in private practice.
2. Nurses of the Farrand Training School were on special duty night and day.
3. Diagnosis of every case was verified by bacteriological examination.
4. Cases were not selected; admitted in all stages of the disease.
5. There were four cases of tracheotomy, two being combined with intubation. All recovered.
6. The use of repeated doses of the serum (P. D. & Co.'s) was of greatest improvement.
7. No case died that was treated within two days of invasion.

8. No case developed laryngeal stenosis after anti-diphtheritic serum was given.

9. No case developed anuria, convulsions or severe hemorrhages.

10. No abscesses.

11. No deleterious effects.

12. None of the cases under treatment became septic.

13. No constant effect on the temperature or pulse. Temperature above 102° usually fell 1 to 5 degrees in fifteen hours.

14. The membrane loses its progressive character under this treatment.

15. No case of heart paralysis or other heart lesion followed.

16. Four cases very suddenly improved.

17. Sixteen cases were above average severity.

18. Albuminuria developed in eight cases. No casts found.

19. Paralysis was not prevented; four cases or 16 per cent. developed. Average, without antitoxin, 10 to 20 per cent.

20. Rash developed in eight cases, quickly disappearing.

21. There were two deaths, or 8 per cent.; one being moribund on admission.

22. One case recovered with typical reaction when injected on the eleventh day of the disease.

23. The serum does not interfere with the use of local disinfectants or antiseptics.

24. Subsidiary local and constitutional treatment must not be neglected.

25. It is a specific remedy when used early.

---

### WATER.

When judiciously taken in half-pint doses as a laxative in the morning, as a sedative at night, as a diuretic when the skin is cool, as a diaphoretic when the skin is warm, as an expectorant or a refrigerant, its value is remarkable.—*Pye-Smith.*

---

### COLD IN THE HEAD.

Gelsemium is the most potent drug known for the relief of this unpleasant condition. If taken early, drop doses of the fluid extract administered hourly usually secure most satisfactory results. —*Medical Summary.*

An equally efficacious procedure is the administration of aromatic spirits of ammonia and of the sweet spirits of nitre—

15 to 20 M. of each—every alternate hour, for twenty-four hours. These remedies have the effect of stimulating the secretions and thus counteract the first symptoms of an ordinary cold and abort it. Try it.—*Reporter*.

---

### INQUIRIES RELATING TO THE COMPARATIVE VALUE OF EXPECTORANTS AND COUGH REMEDIES.

Crook, in the *Medical Record* of February 22, 1896, makes an interesting report on the efficacy of different drugs for the relief of cough, and tabulates his results as follows:

| Remedies Tested. | Results Obtained. | | | | |
|---|---|---|---|---|---|
| | Total Cases. | Success. | Partial Success. | Failure. | Remarks. |
| Aconite | 12 | .. | 1 | 11 | Case of cardiac hypertrophy. |
| Ammon. bromide | 18 | .. | 12 | 6 | Unselected cases. |
| Ammon. carbonate | 40 | 10 | 15 | 15 | " " |
| Ammon. chloride | 60 | 25 | 20 | 15 | " " |
| Antimony | 24 | 8 | 12 | 4 | " " |
| Apomorphine | 22 | 8 | 12 | 2 | Adults of good physique. |
| Chloroform | 12 | 2 | 6 | 4 | Mild bronchial coughs. |
| Codeine | 16 | .. | 16 | .. | Unselected cases. |
| Creosote | 30 | 6 | 24 | .. | Phthisis or suspected phthisis. |
| Glycerin | 12 | .. | 5 | 7 | Unselected cases. |
| Glycyrrhiza | 20 | .. | 20 | .. | " " |
| Hoffman's anodyne | 10 | .. | 5 | 5 | Cases of bronchitis accompanied by asthma, emphysema or cardiac palpitation. |
| Hydrocyanic acid | 24 | .. | 16 | 8 | Unselected cases. |
| Ipecac | 30 | 5 | 20 | 5 | " " |
| Opiates. { paregoric, morphine | 15 | 3 | 10 | 2 | " " |
| Phenacetin | 30 | 8 | 20 | 2 | " " |
| Potassium bromide | 10 | .. | .. | 10 | " " |
| Potassium iodide | 37 | .. | 15 | 21 | " " |
| Squill | 20 | .. | 16 | 10 | " " |
| Sodium chloride | 12 | .. | 8 | 4 | All mild cases of bronchitis or tracheitis. |
| Terebene | 20 | 2 | 14 | 14 | Unselected cases. |
| Terpin hydrate | 15 | .. | 3 | 2 | " " |
| Tolu | 15 | 2 | 10 | 3 | " " |
| Wild cherry | 10 | .. | 4 | 6 | " " |

The author lays no claim to originality in the observation that few cough remedies possess any great value when given alone. The best results are achieved by a scientific combination of two or more remedies, having due regard for their chemical compatibility, their physiological actions and their applicability in individual cases. The following formulas have been thoroughly tested by the writer in hospital and private practice, and may be trusted to render good service in properly selected cases:

1. For irritative coughs :

℞ Phenacetin .......................... gr. xx.–xl.
Ext. glycyrrhizæ ..................... gr. xx.
Sacch. albi .......................... ʒ ij.

Fiat pulvis, in chartulas xx. dividendus. S. One to be taken at one, two or three hour intervals.

2. For same of more obstinate character :

℞ Phenacetin .......................... gr. l.
Ext. glycyrrhizæ ..................... gr. xx.
Codeinæ sulphatis .................... gr. ij.–gr. iv.
Sacch. albi .......................... ʒ ij.

Fiat pulvis, in chartulas xx. dividendus. S. One to be taken at one, two and three hour intervals.

3. When an expectorant effect is desired :

℞ Extracti glycyrrhizæ ................ gr. xx.
Phenacetin ........................... gr. xx.–xl.
Ammonii muriatis ..................... ʒ i.–ij.
Sacch. albi .......................... ʒ ij.

M. et in chart. xx. div. S. One powder to be taken in a little water every two, three or four hours.

4. A good stimulating expectorant for adults :

℞ Apomorph. hydrochloratis ............ gr. i.

Syr. ipecacuanhæ ..................... fl. ʒ ij.

Syr. tolutani ........................ fl. ℥ i.

Aquæ dest .......................q. s. ad fl ℥ iij.

Ft. mist. S. A teaspoonful five times daily at four hour intervals. Shake well before using. Prepare freshly as required and keep in a dark-colored bottle.

5. A good stimulating expectorant for every-day bronchial and phthisical coughs :

℞ Ammonii muriatis .................... ʒ ij.
Tincturæ opii camphoratæ,
Spiritus chloroformi,
Syr. ipecacuanhæ ................. āā fl. ʒ ij.
Syr. prun. virginianæ ...........q. s ad fl. ℥ iij.

M. S. A teaspoonful every three or four hours. Shake well before using.

6. For weak and fruitless coughs with loss of bronchial power:

℞ Ammon. carbonatis.................... ʒ i.–ij.

Tinct. tolutani........................ fl. ʒ ij.

Syr. senegæ,

Spiritus vini gallici,

Syr. simplicis........................āā fl. ʒ iv.

Aquæ destillatæ.................q. s. ad fl. ℥ iij.

Ft. mist. S. A teaspoonful in a little water every two, three or four hours.

7. For asthmatic and emphysematous coughs:

℞ Spiritus ætheris compositi ............ fl. ʒ iv.

Potassii iodidi,

Ammon. muriatis ..................āā ʒ ij.

Codeinæ sulphatis..................... gr. ij.

Syr. tolutani.......................... fl. ʒ iv.

Aquæ des...........................q. s. ad fl. ℥ iij.

M. S. A teaspoonful every two, three or four hours.

8. For recurring bronchitis or winter cough:

℞ Terebene............................. fl. ʒ vi.

Ol. eucalypt. ......................... fl. ʒ ij.

M. S. Ten to fifteen drops on a little sugar every three or four hours.

---

### WATER IN THE TREATMENT OF NEURALGIA.

Dr. Buxbaum, according to the *Charlotte Medical Journal*, first called the attention of the profession to this mode of treating neuralgia. He thinks that the hydrotherapeutic treatment of this disease has hardly received the attention which it deserves. In neuralgia of rheumatic origin it acts by inducing increased blood supply to the affected parts, and in the neuralgia following upon infective diseases, or due to intoxication by mercury or lead, it promotes the elimination of the poison.

He reports that in eighty-three typical cases of neuralgia this treatment was unsuccessful only in 10 per cent., whereas 60 per cent. were cured and the remainder considerably relieved. The alternate application of heat and cold is most to be recommended. The patient is exposed to high temperatures, and afterwards cold applications are made. The alternating Scotch douche is particularly of service. Recent neuralgias may often be cut short in this way. Patients with sciatica treated without effect by various therapeutic measures, even including nerve stretching, have been cured in a short time by this method. If the neuralgia persists it is nearly always due to some irremediable cause, with the exception of some few cases open to operation. If a remission occurs after the treatment has begun, it shows the neuralgia is curable, and is, therefore, of prognostic value. In trigeminal neuralgia, hydrotherapeutic measures applied to the whole body are the most suitable. Of course, other indications should be attended to, such as anemia, malaria, and the like.—*St. Louis Clinique.*

---

### SUMMER DIARRHEA OF CHILDHOOD.

RARDIN, J. S., (*Cincinnati Lancet-Clinic,*) in commenting on the various methods of treatment comes to the following conclusions :

Astringents, which were formerly so extensively used, have very properly been relegated to the waste dump as useless.

1. Summer diarrhea is caused largely by improper and unclean feeding, and is largely preventable.
2. Bacteria plays a very important part in its development.
3. Hot weather has to do only in an indirect manner, as it promotes the growth and development of bacteria in the food-supply.
4. Treatment consists, first, in eliminating all decomposing food from the bowels by cathartics, lavage and colonic irrigation.
5. Drugs, judiciously administered, are of great value, but are secondary in importance to prevention and management.

